# Ubiquitous Dissolved Inorganic Carbon Assimilation by Marine Bacteria in the Pacific Northwest Coastal Ocean as Determined by Stable Isotope Probing

**DOI:** 10.1371/journal.pone.0046695

**Published:** 2012-10-03

**Authors:** Suzanne DeLorenzo, Suzanna L. Bräuer, Chelsea A. Edgmont, Lydie Herfort, Bradley M. Tebo, Peter Zuber

**Affiliations:** 1 Center for Coastal Margin Observation & Prediction and Division of Environmental & Biomolecular Systems, Oregon Health & Science University, Beaverton, Oregon, United States of America; 2 Appalachian State University, Rankin Science South, Boone, North Carolina, United States of America; J. Craig Venter Institute, United States of America

## Abstract

In order to identify bacteria that assimilate dissolved inorganic carbon (DIC) in the northeast Pacific Ocean, stable isotope probing (SIP) experiments were conducted on water collected from 3 different sites off the Oregon and Washington coasts in May 2010, and one site off the Oregon Coast in September 2008 and March 2009. Samples were incubated in the dark with 2 mM ^13^C-NaHCO_3_, doubling the average concentration of DIC typically found in the ocean. Our results revealed a surprising diversity of marine bacteria actively assimilating DIC in the dark within the Pacific Northwest coastal waters, indicating that DIC fixation is relevant for the metabolism of different marine bacterial lineages, including putatively heterotrophic taxa. Furthermore, dark DIC-assimilating assemblages were widespread among diverse bacterial classes. Alphaproteobacteria, Gammaproteobacteria, and Bacteroidetes dominated the active DIC-assimilating communities across the samples. Actinobacteria, Betaproteobacteria, Deltaproteobacteria, Planctomycetes, and Verrucomicrobia were also implicated in DIC assimilation. *Alteromonadales* and *Oceanospirillales* contributed significantly to the DIC-assimilating Gammaproteobacteria within May 2010 clone libraries. 16S rRNA gene sequences related to the sulfur-oxidizing symbionts Arctic96BD-19 were observed in all active DIC assimilating clone libraries. Among the Alphaproteobacteria, clones related to the ubiquitous SAR11 clade were found actively assimilating DIC in all samples. Although not a dominant contributor to our active clone libraries, Betaproteobacteria, when identified, were predominantly comprised of *Burkholderia*. DIC-assimilating bacteria among *Deltaproteobacteria* included members of the SAR324 cluster. Our research suggests that DIC assimilation is ubiquitous among many bacterial groups in the coastal waters of the Pacific Northwest marine environment and may represent a significant metabolic process.

## Introduction

Inorganic carbon assimilation in the euphotic zone is most often attributed to oxygenic photosynthesis. Photosynthetic organisms fix DIC via the Calvin-Benson-Bassham (CBB) cycle, utilizing the CO_2_ fixing enzyme ribulose-1, 5-bisphosphate carboxylase oxygenase (RubisCO) [1]. However, recent research has uncovered widespread bacterial DIC assimilation by mixotrophic organisms and alternative carbon fixation pathways [2], blurring the lines between strict autotrophic and heterotrophic behavior.

Ubiquitous in the euphotic zone, strains of aerobic anoxygenic photosynthetic bacteria, particularly members of the *Roseobacter* clade [3] have been shown to use a mixotrophic carbon metabolism [4]–[6]. Additionally, it has been demonstrated that related strains, including *Rhodobacter sphaeroides* and *Rhodospirillum rubrum,* can fix CO_2_ independently of RubisCO [7]. Furthermore, photoheterotrophic members of the Gammaproteobacteria group NOR5/OM60, such as strain HTCC2080, may even be capable of mixotrophy via the 2-hydroxypropionate cycle [8].

Previous research has demonstrated the importance of CO_2_ assimilation in heterotrophic bacteria, which depends on several factors including 1) the type of organic substrate utilized for growth [9]–[10], 2) the metabolic state of the organisms, and 3) the different bacterial species that show the capability of incorporating CO_2_ to replenish biomass components [11]. For example, Feisthauer et al. [11] noted a high-level, growth phase-independent, labeling of the oxaloacetate-derived amino acids when grown on glucose as the sole carbon source, indicating the occurrence of heterotrophic CO_2_ fixation in *Pseudomonas knackmussii* and the Alphaproteobacterium *Rhodococcus opacus*. Examples of facultative autotrophy and mixotrophy are also present among other Alphaproteobacteria including members of the genera *Rhodopseudomonas, Bradyrhizobium, Nitrobacter, Xanthobacter, Paracoccus, Rhodobacter, Sinorhizobium,* Betaproteobacteria including members of the genera *Hydrogenophaga, Rubrivivax, Cupriavidus, Burkholderia, Xanthanomonas, Thiomonas* (formerly known as *Thiobacillus*) and *Alcaligenes*, the Gammaproteobacterium *Methylococcus capsulatus,* the Firmicute *Halarsenatibacter silvermanii* and the Actinobacterium *Nocardia opaca* [12]–[17]. These cultured strains usually contain the type IC (or IA) subgroup of red-like RubisCO genes [13], and a distinct diversity of the type IC genes have been found in organic rich environments such as soils [18]–[19]. The genes were found to be not only abundant in soils [20] but also highly active [21].

Alternative CO_2_ fixation strategies may also play a role in heterotrophic or mixotrophic DIC assimilation as studies of the RubisCO-lacking Flavobacterium *Polaribacter* sp. MED152 showed light-stimulated CO_2_ incorporation [22] suggesting that DIC uptake might play an important role in the life strategy of this marine heterotroph. It is unclear how important many of the alternative CO_2_ fixation strategies are for *Polaribacter* and other heterotrophic species in marine waters, although Alonso-Sáez et al. [23] demonstrated that heterotrophic CO_2_ assimilation was active within the nutrient-depleted stationary phase conditions in Arctic sea water cultures.

The majority of marine bacteria remain uncultured and the processes governing DIC assimilation are difficult to elucidate. However, Swan et al. [24] reported that RubisCO is encoded in the genomic DNA of uncultured marine bacteria identified through singled-cell sorting and whole-genome amplification. The authors conclude that with the presence of RubisCO in the genomic DNA the potential for chemolithoautotrophy exists among Gammaproteobacteria clusters Arctic96BD-19 and Agg47 and some uncultured *Oceanospirillales*. RubisCO has also been detected in the Alphaproteobacteria *Aurantimonas manganoxydans* [25], which has been found in the Pacific Northwest coastal margin [26] and is present among many other Alphaproteobacteria that may be able to grow mixotrophically or chemoautotrophically under certain conditions [5]. Overall, a growing body of evidence suggests that DIC assimilation in the complex marine environment does not fit squarely in the canonical dichotomy of autotrophy versus heterotrophy.

Here we employ stable isotope probing (SIP) with ^13^C-NaHCO_3_ incubations to examine the contribution of various classes of bacteria to active DIC assimilation in the Pacific Northwest coastal waters at three geographically distinct sites. The Newport Hydroline was sampled 10 km offshore (NH-10) at 80 m in September 2008, March 2009, and May 2010 to examine temporal patterns among DIC-assimilating organisms. In May 2010 NH-10, Columbia River Line 20 km from shore (CR-20), and La Push Line 6 km from shore (LP-6) were sampled to examine spatial variations among DIC-assimilating communities (Fig. S1). The goal of this study was to determine if DIC assimilation is widespread among the heterotroph-dominated marine microbial community and to identify dominant organisms among DIC-assimilating communities in the coastal environment.

## Results

### Comparison of Clone Libraries from ^12^C and ^13^C Bands in NH-10 September 2008 and March 2009 Reveals a Distinct Bacterial Population Actively Assimilating DIC

**Figure 1:**
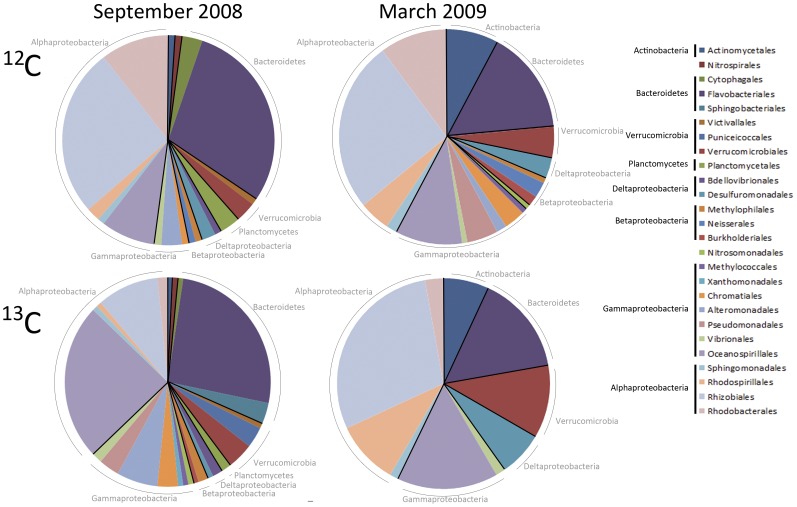
Distribution of bacterial orders observed in 16S rRNA gene clone libraries of ^12^C vs. ^13^C fractions from ^13^C-NaHCO_3_ incubations at NH-10 in September 2008 and March 2009.

In September 2008, Alphaproteobacteria and Bacteroidetes dominated the bacterial assemblage in both ^12^C and ^13^C-labeled fractions, but their distribution between ^12^C and ^13^C bands, respectively, increased from 30% to 38% for Alphaproteobacteria and from 25% to 33%, for Bacteroidetes (Fig. 1). Alphaproteobacteria and Bacteroidetes taxa represented a greater percentage of the actively DIC-assimilating community versus the total assemblage in the coastal ocean in September. In contrast, the distribution of Gammaproteobacteria in the two fractions dropped from 24% in the ^12^C band to only 14% of the active DIC-assimilating community.

Ribosomal Database Project (RDP) naïve Bayesian classifier identified the overall Proteobacteria shift between the ^12^C and ^13^C bands as significant at 2.8^−4^, and within the Gammaproteobacteria at 6.8^−4^ at a 95% confidence threshold for both Proteobacteria and Gammaproteobacteria. When grouped by order, representative Gammaproteobacteria species among the ^12^C band were largely populated by *Oceanospirillales* comprising 21% of all species, but only 8% of the active DIC-assimilating community at that time. Among the Alphaproteobacteria *Rhizobiales* constitute a significant fraction of the active DIC-assimilating community at 25%, but only 9% of species identified in the ^12^C band. *Flavobacteriales* make up a significant portion of both fractions with 28% of the ^12^C band and 25% of the ^13^C band. Shifts between the distributions of the total bacterial population versus the active DIC-assimilating community were observed among less dominant groups, such as the Betaproteobacteria and Verrucomicrobia. Similar shifts were observed between active and inactive fractions among orders within the Bacteroidetes in September 2008 and March 2009 (Fig. 1).

The differences observed between the clone libraries derived from the ^12^C and ^13^C fractions collected from each sample, in terms of taxonomic class and family abundance, suggests that cross-contamination was unlikely. Shifts between the percentage of bacterial communities in the ^12^C vs. ^13^C clone libraries indicate that numerically dominant organisms may not be the most metabolically active in terms of DIC assimilation, supporting observations by Musat et al. [27] who determined that inconspicuous microbes can play a significant role in environmental nitrogen and carbon cycles. For example, while *Rhodobacteraceae* are relatively abundant in both September 2008 and March 2009, however *Flavobacteriaceae* appear to be more active in DIC assimilation (Fig. S2).

A small chloroplast signal was observed within all samples, with the exception of CR-20 at 128 m. The percent diatom contribution to DIC assimilation averaged 12.5% among the remaining May 2010 samples. In March 2009 diatoms contributed only 9% of 16S rRNA sequences to the active fraction. The sole anomaly emerged in September 2008 sample, where chloroplasts accounted for more than 30% of 16S rRNA gene sequences in the library. The focus of the study reported herein is on the bacterial contribution to dark DIC assimilation, and for the purposes of this paper the chloroplast sequences have been removed from the SIP data set.

*Cyanobacteria* represented primarily by *Synechoccocus sp.* 16S rRNA gene sequences, were active in DIC assimilation only in March 2009 at NH-10 and LP-6 in May 2010 contributing no more than 9% of 16S rRNA gene sequences identified.

### Spatial and Temporal Variations in DIC Assimilating Bacteria

**Figure 2:**
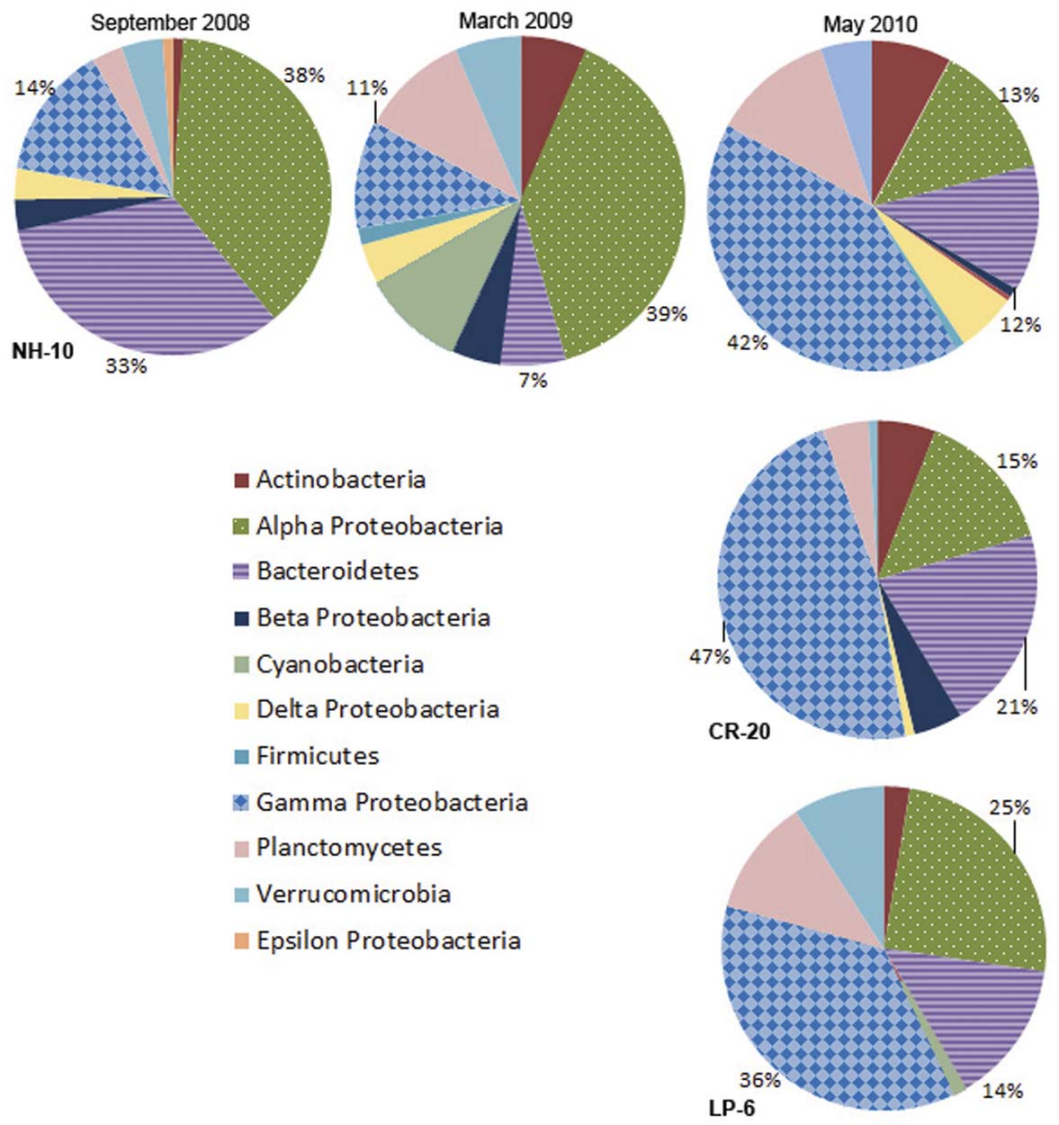
Class distributions of 16S rRNA gene clone libraries of ^13^C fractions from all ^13^C-NaHCO_3_ incubations at NH-10 in September 2008, March 2009, and May 2010, as well as at LP-6 and CR-20 in May 2010.

A variety of taxa were implicated in active DIC assimilation in the northeast Pacific Ocean. Bacterial 16S rRNA gene sequences for DIC-assimilating organisms from the Alphaproteobacteria, Gammaproteobacteria, and Bacteroidetes were abundant at NH-10 in September 2008, March 2009, and at all locations in May 2010 (Fig. 2). Sequences that affiliated with Actinobacteria, Verrucomicrobia, Planctomycetes, and Delta- and Betaproteobacteria were also uncovered from the ^13^C fraction in samples regardless of location, depth, or season, although to a lesser degree than the aforementioned three classes. However the phylogenetic composition within taxonomic groups showed variation at finer taxonomic resolution.

#### Alphaproteobacteria

At NH-10 Alphaproteobacteria were the dominant active DIC-assimilating population in both September 2008 and March 2009, representing 38% and 39%, respectively, of the total assemblage detected in the ^13^C fraction, but formed a significantly smaller portion in May 2010 at 13% (Fig. 2). The reduction in active Alphaproteobacteria 16S rRNA sequences observed in the ^13^C fraction was also observed at the other two sites; 25% and 15% of the ^13^C fractions were found at CR-20 and LP-6, respectively.

**Figure 3:**
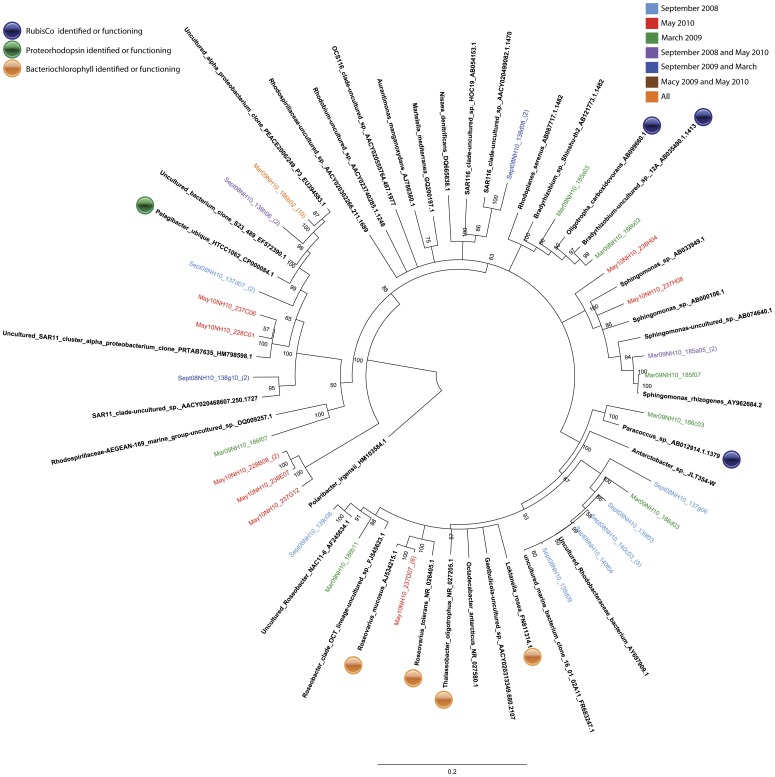
Rooted neighbor-joining phylogenetic tree of *Alphaproteobacteria* species actively involved in DIC assimilation obtained from 16S rRNA gene clone libraries of ^13^C fractions from SIP experiments conducted at NH-10 in September 2008, March 2009, and May 2010. Clones representing highly similar OTUS were collapsed for clarity.

**Figure 4:**
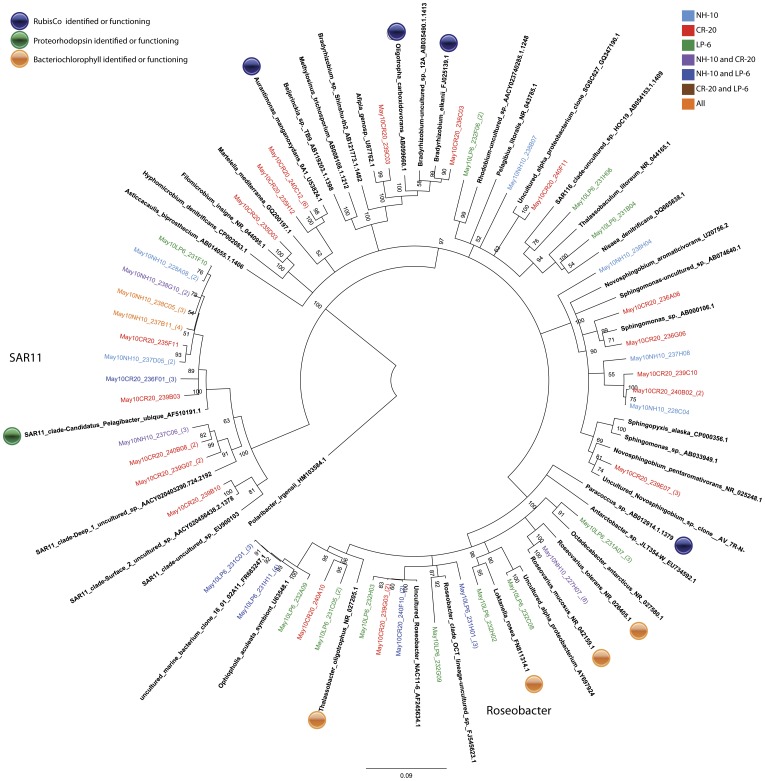
Rooted neighbor-joining phylogenetic tree of *Alphaproteobacteria* involved in DIC assimilation obtained from 16S rRNA gene clone libraries of ^13^C fractions from SIP experiments conducted at NH-10, CR-20, and LP-6 in May 2010. Clones representing highly similar OTUS were collapsed for clarity.

Members of the SAR11 clade comprised a significant portion of the active Alphaproteobacteria assemblage in all samples. Sequences related to the cosmopolitan SAR11 species, *Pelagibacter ubique HTCC1062* were recovered from the ^13^C fractions in the September 2008 and March 2009 samples from NH-10 and in the ^13^C-DNA fractions from all of the May 2010 samples (Fig. 3 and Fig. 4).

Analysis of 16S RNA gene clone libraries constructed from ^13^C-labeled fractions of the NH-10 samples collected in September 2008, March 2009, and May 2010 implicated the *Roseobacter* clade in active DIC assimilation. Among the organisms identified *Roseovarius mucosus* were found only in May 2010 NH-10 and CR-20 samples. This cluster forms 100% of the temporal contribution of the *Roseobacter* clade to DIC assimilation at NH-10 in May and 25% of the overall Alphaproteobacterial assimilation. Members of the *Roseobacter* clade active in DIC assimilation at LP-6 were closely related to *Octadecabacter antarcticus* and *Loktanella rosea*. *Rhizobiales* constituted a significant portion of sequences from the ^13^C-DNA fraction of the CR-20 sample. The most notable sequences within this order were related to *Aurantimonas manganoxydans* and exclusive to CR-20. *Bradyrhizobium* related to *Oligotrophicus carboxidovorans* were detected in the active fraction at NH-10 in May 2010 and March 2009. *Paracoccus sp.* was found to be active in DIC assimilation among the *Rhodobacterales* in March 2009 (Fig. 4).

#### Gammaproteobacteria

At NH-10 the Gammaproteobacteria played a small role in DIC assimilation in September 2008 and March 2009, comprising only 14% and 11% of the active bacterial assemblage, but became the dominant class of DIC-assimilating bacteria (36% - 47%) in all May 2010 clone libraries (Fig. 2).

**Figure 5:**
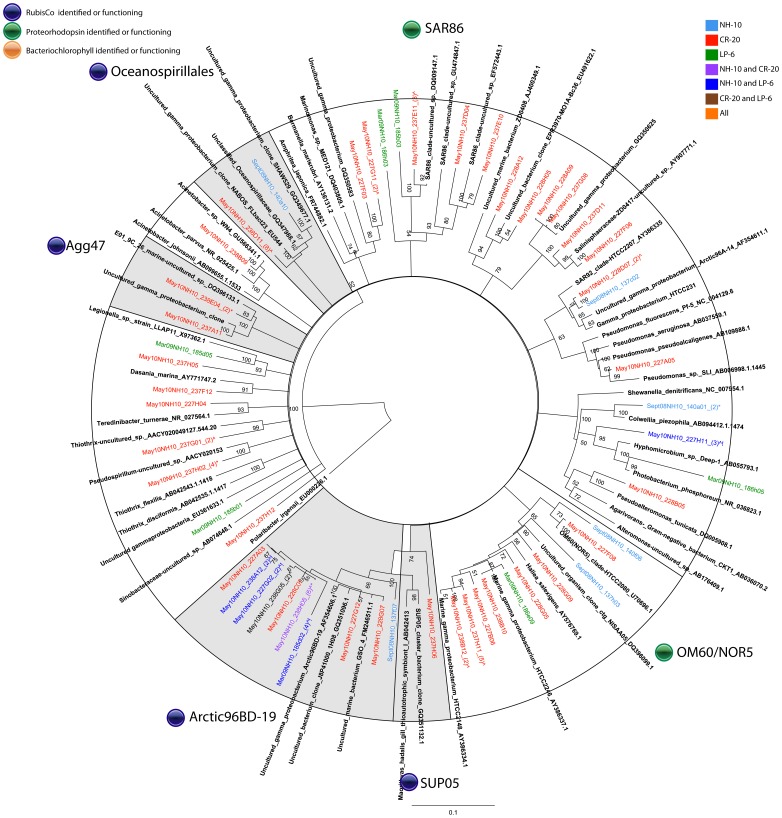
Rooted neighbor-joining phylogenetic tree of *Gammaproteobacteria* involved in DIC assimilation obtained from 16S rRNA gene clone libraries of ^13^C fractions from SIP experiments conducted at NH-10 in September 2008, March 2009, and May 2010. Clones representing highly similar OTUS were collapsed for clarity. Shaded clades represent those identified by Swan et al., 2012 to contain RubisCo and possibly participate in dark carbon fixation.

**Figure 6:**
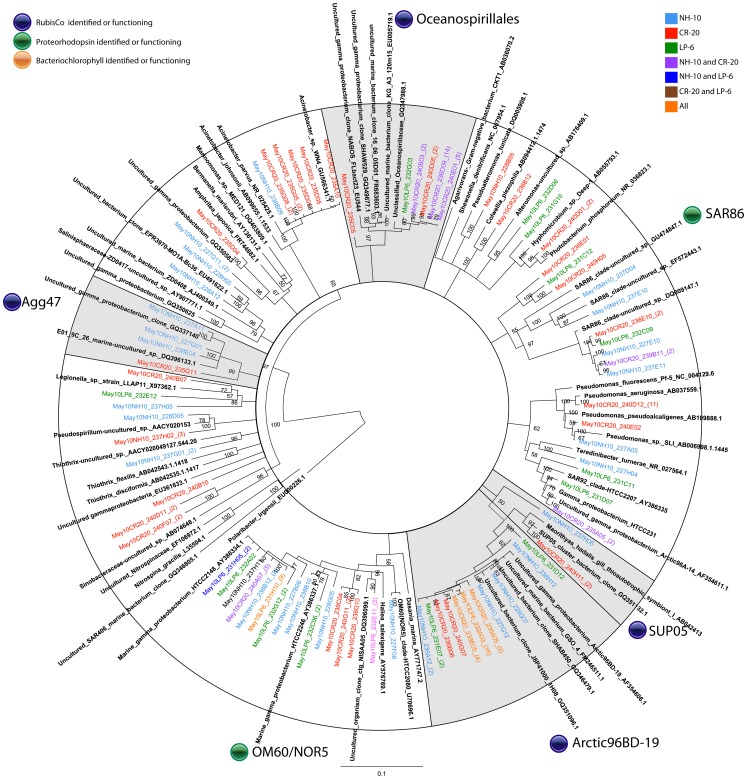
A. Rooted neighbor-joining phylogenetic tree of *Gammaproteobacteria* involved in DIC assimilation obtained from 16S rRNA gene clone libraries of ^13^C fractions from SIP experiments conducted at NH-10, CR-20, and LP-6 in May 2010. Highly similar OTUS were collapsed for clarity. Shaded clades represent those identified by Swan et al., 2012 to contain RubisCo and possibly participate in dark carbon fixation.

In all samples, two orders of bacteria, *Alteromonadales* and *Oceanospirillales,* were most prevalent among the heterotrophic DIC-assimilating community (Figs. 5 and 6). Uncultured organisms related to *Alteromonadales* showed the most even distribution among the three sampling sites. Sequences from these organisms were also found in the March 2009 ^13^C-DNA fraction, but were significantly more dominant among the DIC-assimilating organisms in May 2010. Organisms related to unclassified *Oceanospirillales* were identified among the sequences within the ^13^C-labeled fraction in both September and May samples. Similar to SAR11, this group showed temporal variability, dominant in the May 2010 sample but representing only 7% of the clones within the group in September 2008 (Fig. 5). Sequences of the unclassified *Oceanospirillales* members were the majority at NH-10 and CR-20 in May 2010 with a portion of clones grouping with sulfur oxidizing symbionts, in particular SUP05 and Arctic96BD-10 (Fig. 6).

In the active fraction of May 2010 samples organisms related to *Acinetobacter* and organisms within the Agg47 cluster were only identified at CR-20 and NH-10, although the *Acinetobacter* cluster was more abundant at CR-20 (Fig. 6.). Also identified in DIC assimilation at CR-20 were species closely related to *Pseudomonas aeruginosa;* sequences related to those of *Colwellia piezophila* were identified at NH-10 in September 2008 (Fig. 5).

#### Bacteroidetes

**Figure 7:**
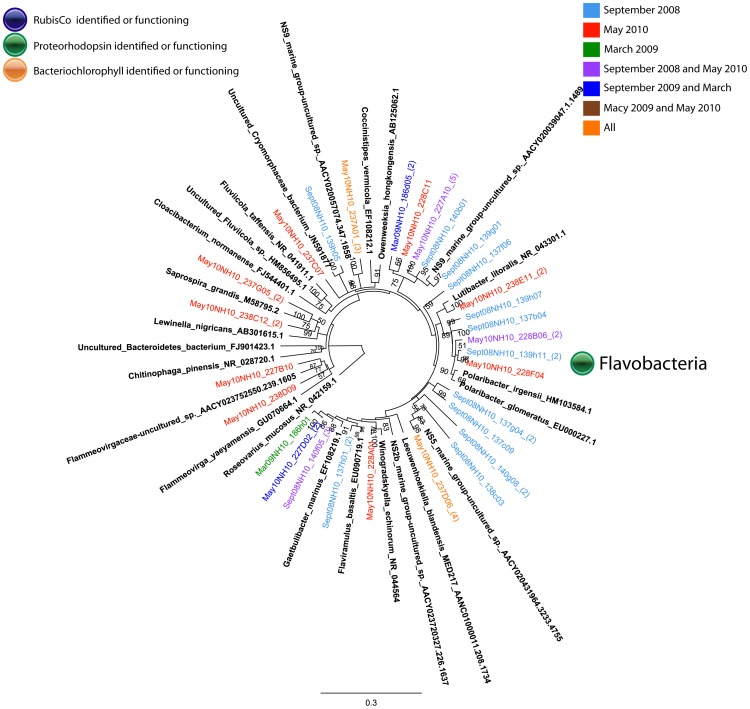
Rooted neighbor-joining phylogenetic tree of *Bacteroidetes* involved in DIC assimilation obtained from 16S rRNA gene clone libraries of ^13^C fractions from SIP experiments conducted at NH-10 in September 2008, March 2009, and May 2010. High similar OTUs were collapsed for clarity.

**Figure 8:**
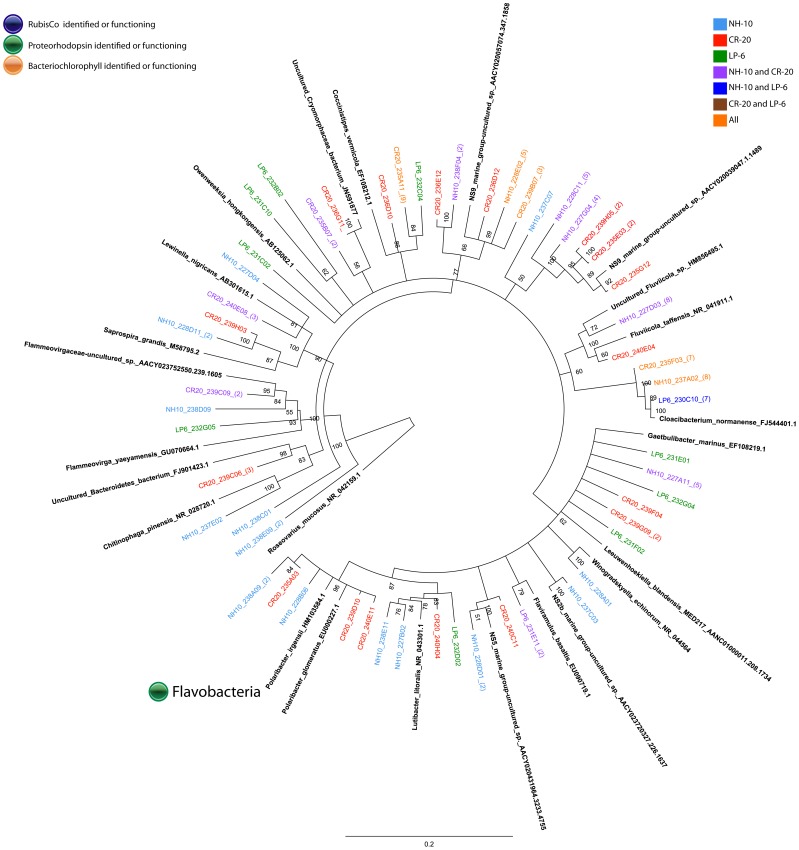
Rooted neighbor-joining phylogenetic tree of *Bacteroidetes* involved in DIC assimilation obtained from 16S rRNA gene clone libraries of ^13^C fractions from SIP experiments conducted at NH-10, CR-20, and LP-6 in May 2010. High similar OTUS were collapsed for clarity.

Sequences representing Bacteroidetes were identified in the ^13^C-fractions of DNA all NH-10 samples (Fig. 2). Bacteroidetes comprised the third largest class at NH-10 (12%) and LP-6 (14%), and the second largest class at CR-20 (21%) active in DIC assimilation in May 2010 (Fig. 2). Analysis of the DIC-assimilating Bacteroidetes assemblage at the species level identified members of the *Flavobacteria*. Sequences specifying *Cloacibaterium normanense* were identified at NH-10 only in May 2010, as well as clones related to *Fluviicola sp.* (also identified in March 2009) (Figs. 7 and 8). Members of the *Polaribacter* clade were active in DIC assimilation at NH-10 in September 2008 and May 2010 and at CR-20 in May 2010. Organisms of the order Sphingobacteriales, related to *Lewinella nigricans* and *Saprospira grandis* were identified in the analysis of the ^13^C-DNA fraction of DNA samples collected at NH-10 and CR-20 in May 2010. Sphingobacteriales, however, were not found to be active in any other samples and were only found in the ^12^C clone libraries from September 2008 and March 2009 (data not shown).

#### Deltaproteobacteria

**Figure 9:**
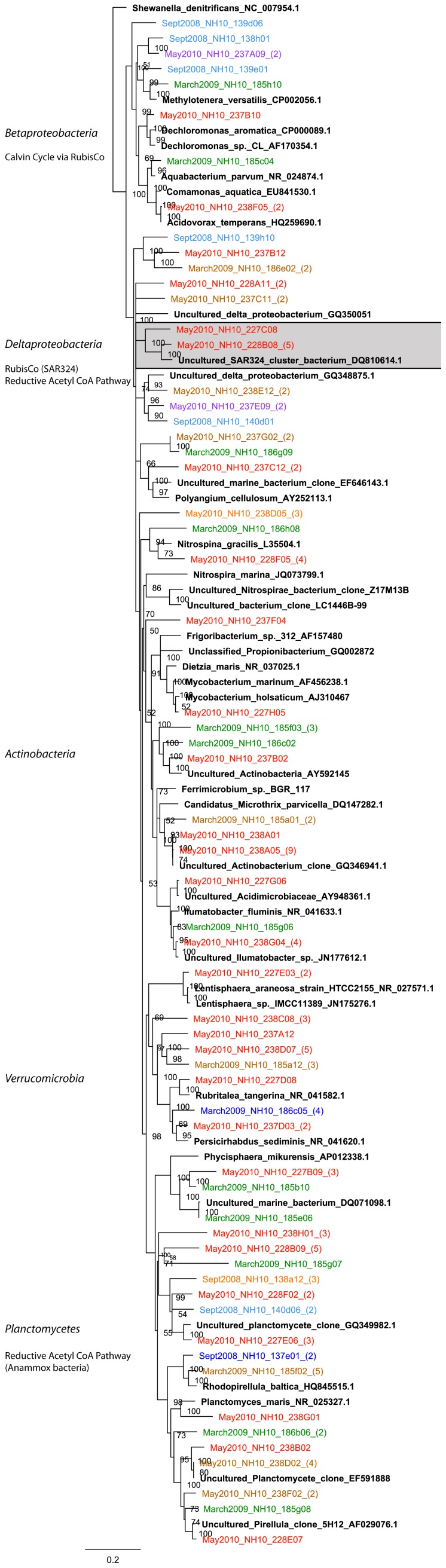
Rooted neighbor-joining phylogenetic tree of Deltaproteobacteria, Betaproteobacteria, Verrucomicrobia, Actinobacteria, and Planctomycetes involved in DIC assimilation obtained from 16S rRNA gene clone libraries of ^13^C fractions from SIP experiments conducted at NH-10 in September 2008, March 2009 and May 2010. High similar OTUs were collapsed for clarity. Shaded clades represent those identified by Swan et al., 2012 to possibly participate in C-1 metabolism or dark carbon fixation.

**Figure 10:**
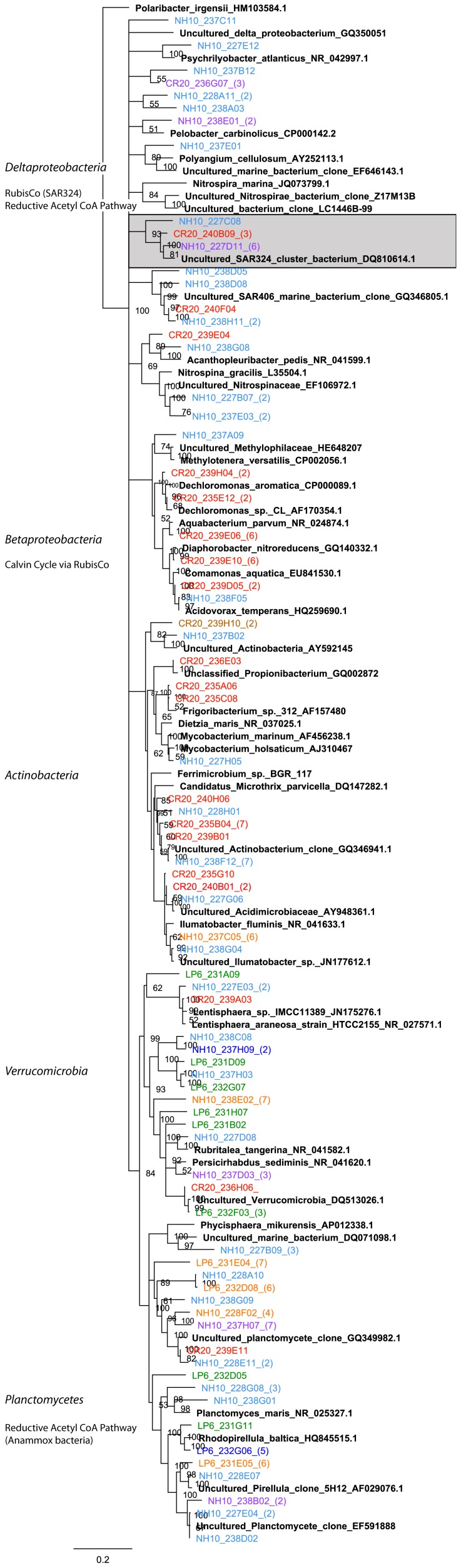
Rooted neighbor-joining phylogenetic tree of Deltaproteobacteria, Betaproteobacteria, Verrucomicrobia, Actinobacteria, and Planctomycetes involved in DIC assimilation obtained from 16S rRNA gene clone libraries of ^13^C fractions from SIP experiments conducted at NH-10, CR-20, and LP-6 in May 2010. High similar OTUs were collapsed for clarity. Shaded clades represent those identified by Swan et al., 2012 to possibly participate in C-1 metabolism or dark carbon fixation.

Deltaproteobacteria contributed to DIC assimilation in the Pacific Northwest Coastal Margin, based on analysis of the ^13^C fraction, but abundance varied across sampling sites and seasons (Fig. 2). Deltaproteobacteria contributed only 2% and 6% respectively of clone library derived from the ^13^C fraction of September 2008 and March 2009 (Fig. 9). SAR324 bacteria were not identified in the March and September clone libraries when the majority of Deltaproteobacteria sequences related to uncultured species. At NH-10 and CR-20 in May 2010 16S rRNA gene sequences in the active fractions were affiliated with the SAR324 cluster and also *Nitrospina sp.* (Fig. 10). *Nitrospina spp.*, however, were active in the March 2009 sample.

#### Betaproteobacteria

Betaproteobacteria represented a small fraction of the active DIC assimilating community overall (Fig. 2). At NH-10 in March 2009 the Betaproteobacteria were predominantly (80%) affiliated with *Acidovorax sp.* and *Aquabacterium sp.* of Burkholderiales (Fig. 9). In May 2010 16S rRNA gene sequences identified at NH-10 were related to *Dechloromonas sp.* A large number of clones identified at CR-20 in May 2010 were related to *Dechloromonas sp.*, *Diaphorobacter nitroreducens*, *Acidivorax temperans*, and *Aquabacterium sp.* (Fig. 10). Betaproteobacteria were not detected in the active fraction at LP-6.

#### Actinobacteria

16S rRNA gene sequences representing Actinobacteria were identified in the ^13^C fraction of all samples (Fig. 2). The majority of sequences affiliated with uncultured environmental clones. Clones in the active fraction of the NH-10 May 2010 samples associated with an uncultured *Actinobacterium* clone distantly related to *Candidatus Microthrix parvicella* (Fig. 9). However, a fraction of the active species identified at NH-10 and CR-20 in May 2010 was closely related to *Ilumatobacter fluminis* (Fig. 10). The contribution of these species to DIC assimilation appears to be more prominent at CR-20 in May 2010 as *Ilumatobacter sp.* accounted for 33% of active Actinobacteria species identified in that clone library.

#### Planctomycetes

Planctomycete species were identified in all active fractions with a number of clones associated with the traditionally heterotrophic genera *Pirellula* and *Rhodopirellula*. Sequences related to those of *Rhodopirellula baltica* were identified within all NH-10 samples (Fig. 9). A few clones also grouped with *Planctomyces maris* in the May 2010 NH-10 samples. Both *Rhodopirellula baltica* and *Planctomyces maris* were identified in the active fraction at LP-6 in May 2010, yet these organisms were not found in the CR-20 clone library (Fig. 10).

#### Verrucomicrobia

Verrucomicrobia were identified in the active fractions of all samples (Fig. 2). A number of these 16S rRNA gene sequences were associated with the order *Verrucomicrobiales*. A small percentage of the sequences from the NH-10 May 2010 and March 2009 clone libraries aligned closely with *Persicirhabdus sediminis* (Fig. 9). 16S rRNA gene sequences related to *Lentisphaera araneosa* and *Rubritalea tangerina* were also identified in the active DIC assimilating fraction at NH-10 and CR-20 in May 2010 (Fig. 10).

## Discussion

### DIC Assimilation- General Considerations

Bacterial 16S rRNA gene sequence data from SIP experiments conducted on water collected from the Oregon and Washington coasts demonstrate that Alphaproteobacteria, Gammaproteobacteria, and Bacteroidetes species are the bacterial classes most active in assimilating DIC into their DNA. Their predominance among DIC assimilating communities is not entirely surprising as the three classes have been identified as dominant in marine waters [28], [29]. Several other bacterial classes such as Betaproteobacteria, Deltaproteobacteria, Verrucomicrobia, Actinomycetes, and Planctomycetes were also observed to participate in DIC assimilation (Fig. 2). Distinguishing between heterotrophic and autotrophic metabolism is difficult as many of the organisms identified within our data sets are uncultured or returned matches to sequences in GenBank from non-marine sources, such as soil and sediment. Our data suggest that prokaryotic DIC assimilation, whether heterotrophic, chemoautotrophic, or mixotrophic, is a more significant process in the coastal environment than previously thought and is, in fact, widespread among marine bacterial classes.

### Methodological Considerations

It is important to note that we cannot entirely rule out the possibility of cross-feeding of ^13^C-labeled substrates between organisms. One of the major caveats to many, if not all, SIP protocols is the possibility of detecting secondary consumption of stable isotope-labeled metabolites produced by other organisms. In our case, the uptake of ^13^C-labeled HCO_3_^−^ during autotrophic carbon fixation could lead to the production of ^13^C-labeled organic compounds. Although carbon fixation can occur within minutes [30], the production of sufficient amounts of organic carbon able to be incorporated into the DNA at a distinguishable level could take hours. In an effort to limit cross-feeding we chose a short incubation time of 3 hours, which is the minimum time required to achieve successful labeling of DNA with ^13^C. While previous studies in coastal sediments were able to discern labeled DNA within 1 hour of incubation with the addition of the *Halobacterium salinarum* carrier DNA to the CsCl gradient [31], we found it difficult to consistently obtain sufficient amounts of labeled DNA for 16S rRNA amplification and cloning from incubations under 3 hours. To the best of our knowledge, our incubation time is the shortest reported for a stable isotope experiment in marine waters. In fact recent SIP publications in similar environments report incubation times ranging from 24 hours [32] to 72 hours [33].

While a short incubation time mitigates the effects of cross-feeding, the possibility that our ^13^C amplification data is merely the result of the buoyant density of DNA affected by G+C content must be directly addressed. Previous research suggests that ^12^C-DNA with high G+C content may co-migrate with ^13^C-labeled DNA, resulting in ^13^C fractions containing isotopically labeled DNA, as well as unlabeled DNA from organisms with a high genome G+C content [34], [35]. In our study we found little evidence of bacterial DNA contamination in the ^13^C band in the unamended environmental samples as well as the environmental samples incubated with 2 mM ^12^C-NaHCO_3_. Q-PCR samples showed only minor, late stage amplification in the ^13^C fraction of control groups when compared to the experimental fraction (data not shown). Efforts to clone 16S rRNA gene from the ^13^C control fractions failed. Furthermore, average G+C content of the 16S rDNA sequences identified in the ^12^C versus ^13^C fractions were nearly identical in the September 2008 and March 2009 samples, varying by only 1% between the fractions (data not shown). Therefore, in good agreement with the results of Gallagher et al. [31] our controls suggest that the variable G+C content in environmental samples did not affect our SIP study that utilized carrier DNA.

### Mechanisms of DIC Assimilation in Marine Bacteria

#### Obligatory and facultative use of RubisCO

Studies of Alonso-Sáez, et al. [23] highlighted the potential for high bicarbonate assimilation among Arctic heterotrophic bacteria. Swan et al. [24] combined single-cell sorting with whole-genome amplification to identify RubisCO and sulfur oxidation genes in a number of Gammaproteobacteria, including uncultured ARCTIC96BD-19 and *Oceanospirillales* as well as the potential for C-1 metabolism among Deltaproteobacteria within the SAR324 cluster. The authors suggest the potential for widespread chemolithoautotrophy among uncultured Proteobacteria lineages in the dark ocean.

Supporting the findings Alonso-Sáez et al. [23] and Swan et al. [24], sequence data from our 16S rRNA gene clone libraries confirmed that the Gammaproteobacteria were actively incorporating DIC in the coastal environment, but their contribution to total DIC assimilation may vary spatially or temporally. A large DIC-assimilating Gammaproteobacteria assemblage dominated with ARCTIC96BD-19 and uncultured *Oceanospirillales* was uncovered in May. Furthermore, the identification of the SAR324 cluster bacteria with the clone libraries of active fractions suggests the potential for DIC assimilation involved in C-1 metabolism in the Pacific Northwest Coastal Margin.

Among the Deltaproteobacteria 16S rRNA associated with the chemoautotrophic species *Nitrospina gracilis*, which uses CO_2_ as its sole source of carbon, was identified (Fig. 10). The majority of remaining Deltaproteobacteria were related to organisms associated with sulfur metabolism such as *Pelobacter sp.*, which have shown the ability to utilize CO_2_ during fermentation processes [36]. This class of bacteria was not active in DIC assimilation at LP-6, possibly due to the location and shallow depth (50 m compared to NH-10 and CR-20, see *Methods*) of the sampling site.

Further potential for DIC assimilation exists among Alphaproteobacteria clones found within the ^13^C-fractions, such as nitrogen-fixing, CO-oxidizing heterotrophs among the *Bradyrhizobia, Oligotropha carboxidovorans*, and *Bradyrhizobium elkanii* are known to contain RubisCO [37] (Fig. 3 and Fig. 4). RubisCO has been identified in *Aurantimonas manganoxydans* and may be capable of CO_2_ fixation under the proper conditions [38].

The detection of Betaproteobacteria *Diaphorobacter sp., Acidovorax sp.,* and *Dechloromonas sp.* suggests that CO_2_ assimilation, possibly through RubisCO, associated with nitrogen cycling may have been occurring among members of this class (Fig. 10). Within some *Burkholderia* species, such as the facultative mixotroph *Burkholderia xenovorans*, genes encoding the enzymes of the Calvin Cycle have been identified; furthermore the potential for CO_2_ assimilation associated with methylotrophic bacteria was also identified within this class (Figs. 9 and 10) [13], [37].

A number of *Mycobacterium* species of Actinobacteria have demonstrated growth through C-1 metabolism. Some species such as *Mycobacterium smegmatis* and *Mycobacterium gordonae* contain genes encoding the enzymes of the Calvin–Benson–Bassham pathway, while *Mycobacterium gastri* has exhibited RuBisCO activity as well [39]. The recent discovery of autotrophic methanotrophy among Verrucomicrobia by *Methylacidiphilum fumariolicum SolV*, further suggests that some methanotrophs may be able to fix CO_2_ through the Calvin Cycle, presumably using CH_4_ mainly as an energy source [40]. A growing body of research suggests the occurrence of alternate CO_2_ fixation pathways across numerous classes and clades leading us to believe that there is still much to be discovered about the metabolism of diverse oceanic bacteria.

#### Proteorhodopsin and Bacteriochlorophyll

Proteorhodopsin and bacteriochlorophyll, identified in a number of marine bacteria, interact with light, converting it into energy for growth and survival. Light-stimulated uptake of CO_2_ has been reported for *Polaribacter* MED152 [22] and light stimulated growth of *Dokdonia sp.* MED134 was observed in media with minimal organic matter [41]. Both studies suggest that light may play an important role in the life and resilience of these organisms. It is also well known that the cosmopolitan SAR11 bacterium *Pelagibacter ubique* expresses proteorhodopsin proteins [42]. It has been suggested that the CO_2_ uptakes stimulated by proteorhodopsin may fuel such processes as anaplerotic carbon fixation in some marine heterotrophic bacteria [22], although these reactions are often balanced by carboxylation reactions resulting in no net gain of carbon. As of yet, the benefit of these light-dependent proton pumps for many marine bacteria remains unknown.

Many of the clones within the ^13^C fractions implicated in heterotrophic DIC assimilation, including a number related to *Polaribacter sp.* (Fig. 7 and Fig. 8), are known to contain proteorhodopsin, for example members of the SAR86 clade of the Gammaproteobacteria (Fig. 5 and Fig. 6). Others contain bacteriochlorophyll, a light harvesting protein similar to proteorhodopsin, found in a number of *Roseobacter* species. Clones identified within the active fractions, among bacteriochlorophyll-containing clades, were related to *Roseovarius mucosus,* the species *Loktanella rosea*, and *Thalassobacter oligotrophus* (Fig. 3 and Fig. 4). Organisms containing proteorhodopsin or bacteriochlorophyll cannot grow autotrophically, but many can grow mixotrophically. The presence of proteorhodopsin and bacteriochlorophyll among clades identified within the active ^13^C fractions allows for the potential energy to fix assimilated CO_2_. Unfortunately the mechanism of CO_2_ assimilation that may be supported by proteorhodopsin and bacteriochlorophyll among these organisms is difficult to ascertain.

#### Facultative methylotrophy

A few clones within the ^13^C fractions grouped with clades containing facultative methylotrophs. Among the Alphaproteobacteria, sequences specifying *Hyphomicrobium sp.* and *Thalassobaculum litoreum* were observed (Figs. 3 and 4). Facultative methylotrophy has been reported among Bacteroidetes, including *Flavobacterium glycines* [43], and occurrences among the Gammaproteobacteria are well documented. Doronia and Trotsenko [44] found that *Pseudomonas* sp. M4 assimilated 2.1% of cellular biomass from CO_2_ when grown on glucose, but 47.1% when grown on methylamine. Ro et al. [45] found that RubisCO was active in *Acinetobacter* sp. strain JC1 DSM 3803 cultures when grown on methanol. *Corynebacterium spp.* have also been implicated in facultative methylotrophy [46], [47].

#### Acetyl-coA Pathway

Among the Planctomycetes the potential for CO_2_ assimilation is evident among members of the anammox bacteria, which can fix inorganic carbon via the acetyl-coA pathway [48]. While Planctomycetes were identified in the active fraction of all samples, anammox bacteria were not detected. The mechanism of DIC assimilation among the organisms potentially implicated in DIC assimilation such as *Planctomycetes maris* remains unknown (Fig. 9 and Fig. 10).

Although it is difficult to ascertain the mechanisms of DIC assimilation for each organism or class identified in our 16S rRNA clone libraries, this research provides a foundation for further inquiry regarding widespread DIC assimilation by bacteria in the marine environment.

### Spatial Distribution of SAR11 and Arctic96BD-19

To gain insight into the spatial distribution of DIC-fixing organisms found within our active clone libraries, a handful of sequences were added to a denoised tag pyrosequence database developed by Crump and Fortunato, 2011 [49]. The OTUs were re-clustered with samples from 329 different water samples taken from the Pacific Northwest coastal margin from 2007 through 2008. Approximately 40% of the bacterial community in ocean samples was represented by two OTUs related to *Pelagibacter ubique* HTCC1062 and the chemoautotrophic organism Arctic 96BD-19. Clone Mar09NH10_186h10, related most closely to *Pelagibacter ubique* HTCC1062 (Fig. 3), accounted for nearly 21% of the total bacterial population identified in the tag pyrosequence database while clone May10LP6_231F03, representing Arctic96BD-19 accounted for 18%. In samples taken from bottom depths, however, Arctic96BD-19 bacteria almost always exceeded the percentage of SAR11 bacteria observed (data not shown).

The third most abundant OTU uncovered in the database was May10NH10_237G09, which resembles unclassified *Oceanospirillales*. These organisms, representing nearly 20% of the active DIC-assimilating *Gammaproteobacteria*, contributed significantly to DIC assimilation in the May 2010 samples but account for only 3.2% of the total bacterial population in the tag pyrosequence database. The organisms appeared in all bottom samples along the coastal shelf regardless of season or location, yet do not appear to participate significantly in DIC assimilation in September 2008, and were absent from the March 2009 clone libraries (Fig. 5). The tag pyrosequencing database results suggest that the major DIC assimilating participants identified in the ^13^C fractions of our SIP clone libraries are found throughout the Pacific Northwest Coastal Margin. However, in the case of the uncultured *Oceansospirillales* clone, DIC assimilating ability does not necessarily correlate with the numerical abundance of an organism in the sample.

### Conclusion

As a survival strategy many marine bacteria are able to undergo shifts in metabolism to adapt to a changing nutritional environment [50] causing some heterotrophic bacteria to utilize alternative pathways of carbon uptake that are characteristic of mixotrophic life styles including facultative chemoautotrophy, mixotrophy, methylotrophy, and other forms of inorganic carbon fixation and anaplerotic reactions. Recent research suggests the potential for DIC fixation among *Oceanospirillales*, and a number of other bacterial families that reside in the mesopelagic ocean [24]. While the actual contribution of these processes to the oceanic carbon cycle is unclear, our results show that bacterial DIC assimilation operates in Oregon coastal waters among a wide variety of microorganisms. The rapidity of DIC incorporation into DNA suggests that this process may involve a variety of assimilation pathways, chemoautotrophic or mixotrophic lifestyles.

## Methods

### Shipboard

Water samples were collected from three geographically distinct coastal ocean transects off the Oregon and Washington coasts during three separate CMOP (Center for Coastal Margin Observation and Prediction) cruises aboard the *R/V Wecoma* (Fig. S1). NH-10 (124.296^o^W, 44.652°N) was sampled 5 meters from bottom depth during cruises on September 12, 2008 to October 10, 2008 (75.5 m, dissolved oxygen (DO) 2.1 mg/L), March 19, 2009 to March 20, 2009 (75 m, DO 2.9 mg/L), and May 20, 2010 to June 6, 2010 (75.5 m, DO 2.9 mg/L). CR-20 (124.453°W, 46.166°N) and LP-6 (124.793°W, 47.917°N) were sampled at 125 m (DO 1.9 mg/L) and 50 m (DO 5.5 mg/L) respectively on the May 20, 2010 to June 6, 2010 cruise only. Water samples were collected with 10-L Niskin sampling bottles attached to a SeaBird CTD (conductivity-depth-temperature) rosette equipped with an O_2_ sensor.

Samples were dispensed under low light conditions into 3 L amber Nalgene bottles, amended with 2 mM unlabeled NaHCO_3_, 2 mM ^13^C-NaHCO_3_, or no additional carbon source, and gassed with N_2_ to in situ oxygen levels as recorded with the CTD O_2_ sensor. Bottles were immediately incubated in the dark at 7°C for 3 hours. Immediately following incubation, water was filtered through a 0.2-µm pore-size Sterivex filter (PES, ESTAR, Millipore) using a peristaltic pump, then fixed with 2 mL RNAlater (Ambion) to preserve DNA and frozen at −80°C.

### Laboratory Analysis

(Fig. S3).

#### DNA extraction

DNA was extracted using a phenol chloroform method previously described by Herfort et al. [51].

#### Stable isotope probing

Stable isotope probing employing *Halobacterium salinarum* (strain *ATCC* 29341/DSM 671/R1) DNA as a ^13^C carrier was performed as previously described by Gallagher et al. [31]. The *H. salinarum* used for visualization of ^13^C-DNA was grown in a ^13^C-labeled ISOGRO powder growth medium (Isotec, Miamisburg, OH). The stable isotope enriched medium was prepared for Van Niel’s media amended with 25% NaCl for halophilic bacteria, which were grown aerobically at 25°C for approximately 20 days before the cells were harvested. DNA was extracted as described above.

Approximately 300 ng of environmental sample DNA and 300 ng of ^13^C carrier DNA were added to a 500-µl CsCl density gradient (1 g/ml) containing 20 µg ethidium bromide. The ^12^C and ^13^C fractions were resolved by centrifugation in a TLA 100 rotor on a Beckman ultracentrifuge (Palo Alto, CA) at 225,000×*g* for 24 hours. The bands were then visualized by UV light and were withdrawn from the gradient by first removing the ^12^C-DNA band, changing the pipette tip, releasing a small air bubble above the height of the ^12^C-DNA band, and subsequently removing the ^13^C-DNA band from the gradient. The DNA was then suspended in 200 µl of 10 mM Tris-HCl, 0.5 mM EDTA and cleaned using Millipore Montage PCR Filter Units (Millipore Corp, USA) as per manufacturer’s protocol.

#### Cloning and sequencing of 16S rRNA genes

Cleaned DNA was PCR amplified in triplicate using universal bacterial 16S rRNA gene primers, 27f and 1492r [52]. The cycling conditions were 94°C for 5 min, followed by 30 cycles of 94°C for 1 min, 55°C for 1 min, 72°C for 1 min, with a final extension at 72°C for 10 min. Triplicate 16S rRNA gene PCR products were the pooled and ligated with TOPO vector (pCR 2.1, Invitrogen) and introduced by transformation into One Shot Top 10 electrocompetent *E. coli* cells from the TA cloning kit (Invitrogen). Transformants were inoculated on LB agar supplemented with 40 µg/ml XGAL (bromo-chloro-indolyl-galactopyranoside) and 50 µg/ml ampicillin and grown overnight at 37°C. Positive (white) colonies were picked randomly and transferred into 96-well plates containing 150 µl of 2× Yeast Extract/Tryptone amended with 50 µg/ml ampicillin and incubated overnight at 37°C with shaking at 50 rpm. Following the addition of 30 µl of 50% glycerol, the 96-well plates were then stored at −80°C and sent either to the Genome Sequencing Center at Washington University in St. Louis (September 2008 and March 2009 samples) or Beckman Coulter Genomics (May 2010 samples) for sequencing with primers M13f, M13r, 704f, and 926r to obtain full length 16S rRNA gene sequences. Both facilities employ capillary sequencing using the Big Dye protocol (Applied Biosystems).

#### Control samples: phylogenetic analysis and comparison of 12C vs. 13C bands

To examine differences between the ^12^C and ^13^C DNA fractions extracted from ^13^C-HCO_3_^−^, bacterial 16S rRNA gene clone libraries were constructed for both fractions from water collected in September 2008 and March 2009 on the Oregon coast at NH-10. For both of these samples, large differences in bacterial composition at both class and species levels could be discerned between ^12^C and ^13^C labeled DNA fractions, and the Bray-Curtis indices of similarity between the ^12^C and ^13^C fractions varied from 10.37 to 14.5 out of 100 for all samples analyzed. These population differences allowed us to compare the total microbial community of the water samples with actively DIC-assimilating communities and to rule out significant band cross-contamination between the ^12^C and ^13^C fractions.

^12^C and ^13^C fractions recovered from the SIP environmental samples amended with 2 mM unlabeled NaHCO_3_, 2mM ^13^C-NaHCO_3_, or no additional carbon source for each season and/or location were subjected to qPCR with universal bacterial 16S rRNA gene primers, 27f and 519r [52]. The amplification protocol consisted of an initial denaturation of 94°C for 3 min, 40 cycles of amplification at 94°C for 30 s, 52°C for 30 s, and 70°C for 30 s followed by a terminal extension step of 72°C for 5 min. Bacterial 16S rRNA gene quantities were standardized using almost full-length amplicons of the Arctic 96BD-19 16S rRNA gene obtained from cloning of environmental DNA obtained in this study.

#### Sequence analysis

Trimmed sequences were assembled into full length 16S rRNA contigs using Geneious Pro 5.3.4 [53]. Sequence contigs were then aligned using the greengenes.lbl.gov [54] Nearest Alignment Space Termination (NAST) program. Chimeras were identified using the online program Bellerophon [55] and subsequently removed from analysis. A phylogenetic tree of 16S rRNA sequences was constructed with the remaining sequence contigs against the SILVA database [56] in ARB [57]. Sequences identified as eukaryotic chloroplasts were removed from the clone libraries. In ARB, nearest neighbors were identified and their sequences were acquired from NCBI: BLAST. The sequence alignments were transferred back into Geneious Pro 5.3.4 for phylogenetic analysis of active bacterial classes. Over all seasons 2,880 bacterial clones were screened resulting in 1,780 quality contigs. During analysis 430 clones were identified as eukaryotic and removed from the dataset (Table S1). Bray Curtis indices of similarity [58] and Shannon indices [59] were calculated by season and location using the Plymouth Routines in Multivariate Ecological Research (PRIMER) software version 6 (PRIMER/E Ltd, UK) program (Table S2).

## Supporting Information

Figure S1 Map of sampling locations along the Pacific Northwest Coastal Margin.
(TIF)

Figure S2 Relative changes in the contribution of individual clones to the September 2008 and March 2009 the clone libraries are represented as dimensionless enrichment factors. The enrichment factor was calculated by dividing the relative abundance of a clone by the relative abundance of the total respective clones in the clone library. Values less than 1 (purple) indicate depletion and values greater than 1 (blue) indicate enrichment of the particular group in the heavier fraction.
(TIF)

Figure S3 Diagram of experimental methods from field sampling through DNA sequencing.
(TIF)

Table S1 Total number of clones screened, number of contigs acquired, eukaryotic contigs removed from analysis and the final number bacterial 16s rRNA sequences analyzed are listed by location and season.
(TIF)

Table S2 Bray-Curtis Similarity Index calculated for Alphaproteobacteria, Gammaproteobacteria, and Bacteroidetes in September 2008, March 2009, and May 2010 samples at NH-10 and NH-10, CR-20, and LP-6 in May 2010.
(TIF)

Supporting Materials S1. (DOC)
