# Supplementary figures and images for: Ubiquitous Dissolved Inorganic Carbon Assimilation by Marine Bacteria in the Pacific Northwest Coastal Ocean as Determined by Stable Isotope Probing
Source: PLoS One. 2012 Oct 3;7(10):e46695. doi: 10.1371/journal.pone.0046695 (PMC3463544; doi:10.1371/journal.pone.0046695)

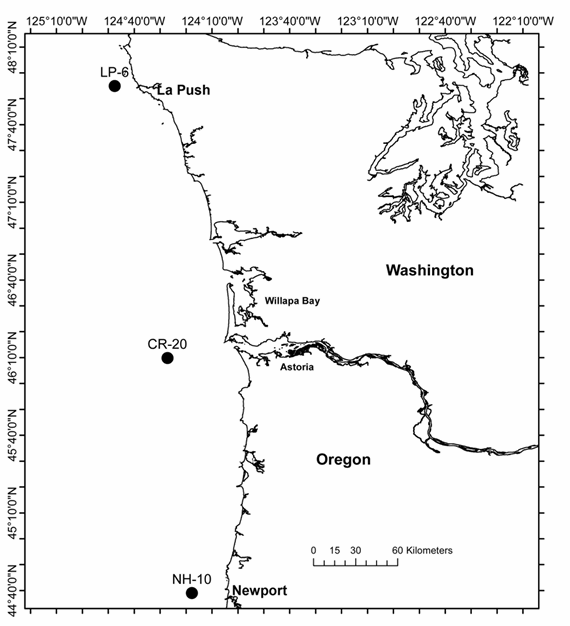

Supplement: Figure S1 — Map of sampling locations along the Pacific Northwest Coastal Margin. (TIF) [file pone.0046695.s001.tif]

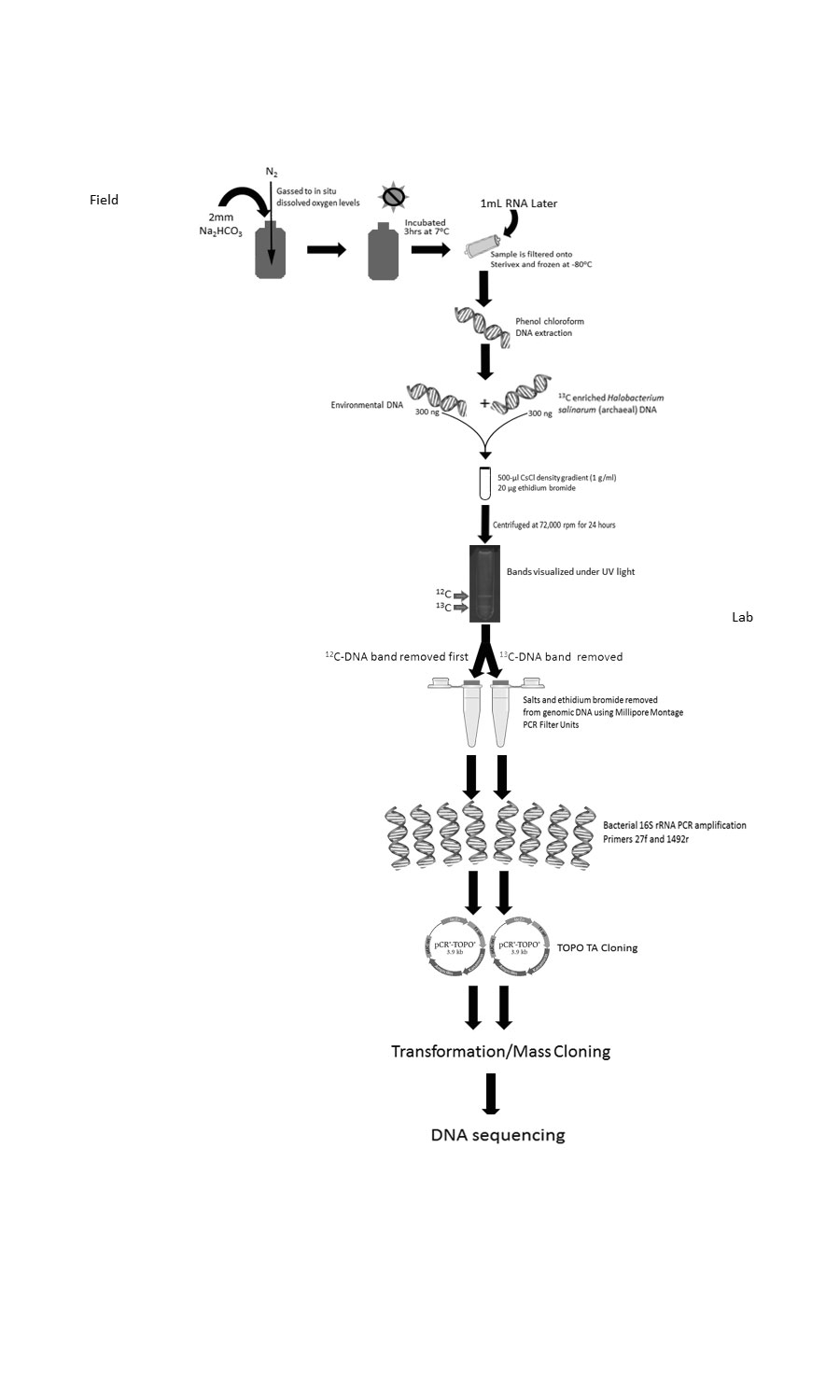

Supplement: Figure S3 — Diagram of experimental methods from field sampling through DNA sequencing. (TIF) [file pone.0046695.s003.tif]
