# Supplementary material for: Ubiquitous Dissolved Inorganic Carbon Assimilation by Marine Bacteria in the Pacific Northwest Coastal Ocean as Determined by Stable Isotope Probing
Source: PLoS One. 2012 Oct 3;7(10):e46695. doi: 10.1371/journal.pone.0046695 (PMC3463544; doi:10.1371/journal.pone.0046695)
Supplement: Supporting Materials S1. — (DOC) [file pone.0046695.s006.doc]

**Supplemental Material For: *Ubiquitous dissolved inorganic carbon assimilation by marine heterotrophic bacteria in the Pacific Northwest coastal ocean as determined by DNA-based stable isotope probing***

Suzanne DeLorenzo^1*^, Suzanna L. Bräuer^1, 2^, Chelsea A. Edgmont^1^, Lydie Herfort^1^, Bradley M. Tebo^1^, Peter Zuber^1^

*^1^Center for Coastal Margin Observation & Prediction and Division of Environmental & Biomolecular Systems, Oregon Health & Science University, 20000 NW Walker Road, Beaverton, OR 97006, USA*

*^2^Appalachian State University, Rankin Science South, 572 Rivers Street, Boone, NC 28608-2027, USA*

*To whom correspondence should be addressed:

*Center for Coastal Margin Observation & Prediction and Division of Environmental & Biomolecular Systems, Oregon Health & Science University, 20000 NW Walker Road, Beaverton, OR 97006, USA.*

Office: (503) 748-1643 Fax: (503) 748 1464 E-mail: [delorenz@ebs.ogi.edu](mailto:delorenz@ebs.ogi.edu)

**Results:**

**Comparison of clone libraries from ^13^C and ^12^C bands in NH-10 September 2008 and March 2009 reveals a distinct bacterial population actively assimilating DIC**

In March 2009 the proportion of the bacterial DIC-assimilating assemblage represented by Alphaproteobacteria, Bacteroidetes, and Gammaproteobacteria decreased. At that time, Alphaproteobacteria comprised approximately 45% of the total bacterial community but accounted for 39% of the active DIC-assimilating bacteria. A similar pattern was apparent for the Bacteroidetes (12% of total bacterial community) and the Gammaproteobacteria (16% of total bacterial community) with these classes accounting for only 6% and 10% of their respective DIC-assimilating community.

Differences between the ^12^C- and ^13^C DNA fractions within the Alphaproteobacteria are dramatic. The ^12^C fraction is dominated in September by members of the family Rhodobacteraceae which make up approximately 51% of the total Alphaproteobacteria, yet only 28% of the active DIC-assimilating community in the ^13^C fraction. The members of the family Aurantimonadaceae made up only 4% of the ^12^C DNA clones representing Alphaproteobacteria, but are the largest group of active DIC-assimilating members of the Alphaproteobacteria at approximately 31%. Between the two DNA fractions from samples collected in March 2009, the family Aurantimonadaceae makes up only 3% of the active DIC-assimilating community but 23% of the total Alphaproteobacteria community, as revealed by analyzing the ^12^C-labeled fraction. Similar shifts were observed among taxa between active and inactive fractions within the Bacteroidetes in September 2008 and March 2009.

If cross contamination occurred during ^12^C and ^13^C band recovery in either sample, comparison of ^12^C versus ^13^C clone libraries would show more similarity between the two fractions. To further refine the comparison of active vs. inactive fractions among the clone libraries a dimensionless enrichment factor was calculated to examine relative changes in the contribution of individual clones to the September 2008 and March 2009 the clone libraries (Fig. S3). In both samples, Flavobacteriaceae represent a significant portion of clones identified in the ^12^C and ^13^C bands suggesting that the majority of the family is active in DIC assimilation, in contrast however, Rhodobacteraceae are relatively abundant in ^12^C fraction in September 2008 but less so in ^13^C fraction. In March 2009, Planctomycetaceae are relatively more abundant in the enriched fraction lending credence to the potential that the most numerically dominant organisms in a system may not be the most metabolically active.
